# Normal values of MAPSE and TAPSE in the paediatric population established by cardiovascular magnetic resonance

**DOI:** 10.1007/s10554-021-02415-9

**Published:** 2021-09-28

**Authors:** Filippo Puricelli, Sabiha Gati, Winston Banya, Piers E. F. Daubeney, Dudley J. Pennell, Inga Voges, Sylvia Krupickova

**Affiliations:** 1 Royal Brompton Hospital, Sydney Street, London, SW3 6NP UK; 2 National Heart and Lung Institute, Imperial College, London, UK; 3 University Hospital Schleswig-Holstein, Campus Kiel, Kiel, Germany

**Keywords:** Cardiovascular magnetic resonance, Children, Normal values, TAPSE, MAPSE

Measurement of mitral and tricuspid annular plane systolic excursion (MAPSE, TAPSE) by echocardiography provides additional information for biventricular functional assessment with high reproducibility. To date, cardiovascular magnetic resonance (CMR) equivalents of MAPSE and TAPSE for paediatric patients are not available. This study aimed to provide a set methodology for the evaluation of longitudinal function and provide the imaging community with normal reference values of CMR-derived MAPSE and TAPSE in healthy paediatric cohorts.

The study population consisted of all children and adolescents retrospectively selected from who underwent a CMR scan between 2016 and 2020, resulting in normal cardiac findings. Images were obtained with 1.5 T scanners (Sonata/Avanto, *Siemens Medical Solutions*, Germany) and were analysed by a single observer using CMR post-processing software (CMRTools; *Cardiovascular Imaging Solutions*, UK).

Lateral and septal MAPSE (L-MAPSE, S-MAPSE) and TAPSE were measured from 4-chamber bSSFP cines. L-MAPSE and S-MAPSE were measured as the distance between the cutting edge of mitral annulus with left ventricular (LV) lateral wall or septum, respectively, captured at end-diastole and at end-systole. TAPSE was measured as the distance between the cutting edge of the tricuspid annulus with right ventricular (RV) free wall captured at end-diastole and at end-systole (Fig. 1).

**Fig. 1:**
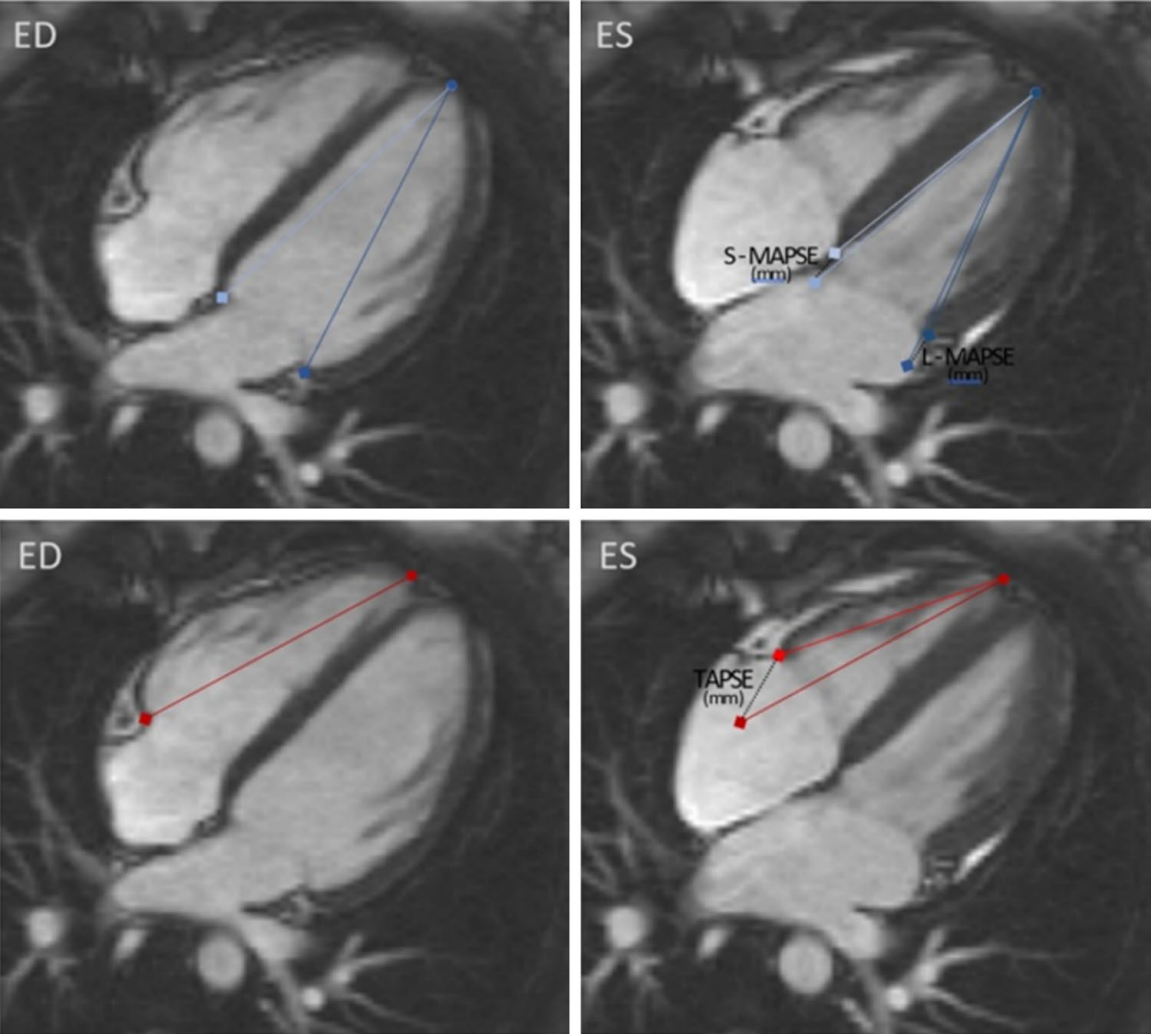
MAPSE and TAPSE measurement using a four-chamber cine image. MAPSE was measured as the distance between the cutting edge of mitral annulus with LV lateral wall (L-MAPSE) or septum (S-MAPSE) captured at end-diastole (ED) and at end-systole (ES). TAPSE as the distance between the cutting edge of tricuspid annulus with RV free wall captured at end-diastole and at end-systole

Simple linear regression was used to establish the association between MAPSE or TAPSE with age, gender, body surface area (BSA), heart rate, ventricular end-diastolic volume index (LVEDVi, RVEDVi) and ejection fraction (LVEF, RVEF). Multiple linear regression was than performed including variables that were significant in the univariate analysis. Intra-observer and inter-observer variability was determined in 25 patients measured by two independent observers, using intraclass correlation coefficient (ICC). All tests were two tailed and significance was set at 0.05.

Fulfilling the inclusion criteria were 138 paediatric patients, of which 88 males, with a mean (SD) [range] age of 13.3 years (2.9) [4.0–17.0]. The mean body surface area (BSA) was 1.6 m^2^ (0.3) [0.7–2.2]. We established three age groups (4–9, 10–14 and 15–17 years respectively), looking for normal distributions.

Table 1 shows normal MAPSE and TAPSE values for age groups.

**Table 1:** Normal MAPSE, TAPSE values for our study population presented in age groups as mean excursion in mm (±1SD) and lower and upper limits

	Age group (yrs)	n. of patients	Mean excursion in mm (±﻿1*SD*)	Lower limit in mm (corresponding centile; z-score)	Upper limit in mm (corresponding centile; z-score)
L-MAPSE	4–9	15	13.3 (±﻿1.3)	10.8 (2.5th; − 2.5)	15.5 (97.5th; + 1.6)
	10–14	66	13.6 (±﻿1.2)	10.0 (2.5th; − 3.2)	15.7 (﻿9﻿7﻿.﻿5﻿t﻿h﻿;﻿ + 1.6)
	15–17	57	14.0 (±﻿1.0)	11.9 (2.5th; − 1.6)	15.9 (﻿9﻿7﻿.﻿5﻿t﻿h﻿;﻿ + 1.9)
S-MAPSE	4–9	15	11.7 (±﻿1.4)	9.5 (2.5th; − 2.6)	13.8 (﻿9﻿7﻿.﻿5﻿t﻿h﻿;﻿ + 0.7)
	10–14	66	12.8 (±﻿1.2)	10.1 (2.5th; − 2.1)	14.9 (﻿9﻿7﻿.﻿5﻿t﻿h﻿;﻿ + 1.6)
	15–17	57	13.2 (±﻿1.3)	9.7 (2.5th; − 2.4)	15.8 (﻿9﻿7﻿.﻿5﻿t﻿h﻿;﻿ + 2.1)
TAPSE	4–9	15	22.8 (±﻿3.8)	18.2 (2.5th; − 1.9)	29.5 (﻿9﻿7﻿.﻿5﻿t﻿h﻿;﻿ + 1.6)
	10–14	66	24.3 (±﻿2.8)	18.6 (2.5th; − 1.8)	29.4 (﻿9﻿7﻿.﻿5﻿t﻿h﻿;﻿ + 1.6)
	15–17	57	24.8 (±﻿3.4)	18.6 (2.5th; − 1.8)	32.7 (﻿9﻿7﻿.﻿5﻿t﻿h﻿;﻿ + 2.6)

For L-/S-MAPSEs, simple linear regression showed a weak but significant correlation with age^2 (r = 0.28, p = 0.001/r = 0.36, p = 0.001), BSA (r = 0.26, p = 0.001/r = 0.32, p = 0.001) and LVEDVi (r = 0.24, p = 0.003/r = 0.22, p = 0.009) but no significant correlation with LVEF. When tested in multivariable model, only correlation with age^2 for both MAPSEs and with LVEDVi for L-MAPSE was maintained.

For TAPSE, simple linear regression showed a weak but significant correlation with age^2 (r = 0.22, p = 0.008), BSA (r = 0.30, p = 0.0001) and RVEDVi (r = 0.33, p = 0.0001) but no significant correlation with RVEF or LVEF. In multivariable model, only correlation with RVEDVi was maintained.

The methods for measuring MAPSEs and TAPSE demonstrated excellent intra-observer agreement (L-MAPSE ICC = 0.88, p < 0.0001; S-MAPSE ICC = 0.85, p < 0.0001; TAPSE ICC = 0.98, p < 0.0001) and high inter-observer agreement (L-MAPSE ICC = 0.71, p < 0.0001; S-MAPSE ICC = 0.81, p < 0.0001; TAPSE ICC = 0.95, p < 0.0001). The mean time to repeat intra-observer measurements was 21 days [13–38].

Ejection fraction (EF) is a widely recognised index of global systolic function in guidelines and clinical practice. However, EF alone has low sensitivity in detecting early contractile impairment with limitations in certain conditions [1].

It is widely considered that the more longitudinally oriented subendocardial fibres provide a significant contribution to normal cardiac function. Strain imaging enables quantification of longitudinal contractile function and has been proven to improve the identification of subclinical LV systolic dysfunction in patients with heart failure with preserved EF (HFpEF). However, compared to echocardiography, CMR-derived longitudinal strain analysis is time consuming and limited by a lack of validation and general availability.

Also MAPSE and TAPSE have been shown to be more sensitive than EF in certain settings including HFpEF. TAPSE showed high negative predictive power in detecting RV dysfunction and has been shown to be an independent predictor of cardiovascular death in the general population, especially in individuals with normal EF [2]. As with echocardiography, CMR-derived TAPSE and MAPSE can be readily available and may serve as additional parameters for the evaluation of LV and RV global function. Because the amount of annular displacement is likely to be affected by heart size and considering growth changes in the paediatric population, it is reasonable not to use a single value but rather reference values to age and/or BSA to best interpret the results. In our study, MAPSE and TAPSE showed only a weak positive correlation with age and ventricular volumes. This weak correlation may be due to the distribution of our study population, as children < 10 years of age are less likely to be referred for a CMR scan than adolescents (who make up almost half of our study population), due to the need for sedation.

## Data Availability

Authors have full control of all primary data and agree to allow the journal to review their data if requested.
